# Multidimensional Health Groups and Healthcare Utilization Among Elderly Chinese: Based on the 2014 CLHLS Dataset

**DOI:** 10.3390/ijerph16203884

**Published:** 2019-10-14

**Authors:** Linglong Ye, Jiecheng Luo, Ben-Chang Shia, Ya Fang

**Affiliations:** 1 School of Public Health, Xiamen University, Xiang’an South Road, Xiamen 361102, China; leyloria@gmail.com (L.Y.); hyzworld@live.com (J.L.); 2 School of Economics, Xiamen University, 422 Siming South Road, Xiamen 361006, China; 3 School of Management, Taipei Medical University, 250 Wuxing Street, Taipei 11031, China; stat1001@tmu.edu.tw

**Keywords:** aging, healthcare utilization, heterogeneity, multidimensional health, person-centered approach

## Abstract

Based on multidimensional health, we aimed to identify health groups among the elderly Chinese population, and examine its relationship with socio-demographic factors on healthcare utilization. Chinese Longitudinal Healthy Longevity Survey in 2014 was adopted. For 2981 participants aged ≥65 years, without missing any health indicators, latent class analysis was adopted to identify health groups. For 1974 participants with complete information, the two-part model was used to assess how health groups and socio-demographic characteristics influence the outpatient and inpatient expenditure. Four health groups were identified and labeled as “Lacking Socialization” (10.4%), “High Comorbidity” (16.7%), “Severe Disability” (7.8%), and “Relative Health” (65.1%). Compared with the relative health group, the lacking socialization group cost higher inpatient expenditure (*p* = 0.02). Those in the high comorbidity and severe disability groups were more likely to use healthcare services and cost higher outpatient expenditure (*p* < 0.01 for all). The effects of socio-demographic factors were also discussed. The findings enhanced our understanding of the heterogeneity of multidimensional health status and complex healthcare demands in the elderly Chinese population. Moreover, it is valuable for improving the allocation of healthcare resource targeted for different groups of the ageing population.

## 1. Introduction

The ageing population has been one of the major challenges of healthcare services in China. There were approximately 229 million (16.2%) Chinese people aged ≥60 years in 2017, and this number is projected to reach 479 million in 2050 [1]. The increase in the elderly population has led to significant health transitions and extensive utilization of healthcare resources. Elderly people are at high risk of suffering from chronic diseases and functional disabilities, and consume the disproportional share of healthcare resources [2,3,4,5]. Thus, there is an increasing concern on the complex health status of the growing elderly population and its demand for healthcare services.

To compare the differences in the health status of the elderly population, most studies generally considered the indicators involving specific chronic diseases, difficulties with the activities of daily living (ADLs), memory impairments, and so on [5,6,7,8]. According to the World Health Organization (WHO), which defines “health” as a state of complete well-being, synthesized by not only physical and psychological dimensions, but also social dimension [9], these health indicators could be assigned to physical and psychological dimensions, but rarely related to the social dimension. Some studies still regard the indicators of social dimension as the influencing factors of health, rather than the composition of health itself [10,11]. However, the unhealthy social status, such as social isolation, has been a critical problem for the elderly. Therefore, with a progressively ageing population, there is an urgent need to comprehensively evaluate the holistic health status of the elderly by integrating health indicators in all three dimensions.

Previous studies have usually adopted variable-centered approaches in assessing the health status of elderly populations. With the assumption that the study population was homogeneous, these approaches focused on describing the associations among observed health indicators [12,13]. However, the potential heterogeneity may exist between individuals with unobserved health indicators, which may result in different patterns of healthcare services utilization. As compared to variable-centered approaches, person-centered approaches focus on the relationships between the individuals and further classifies them into distinct groups, to deal with the heterogeneity [12]. Latent class analysis (LCA), a person-centered approach, can identify the unobservable groups and explore the probabilities of multiple observed health indicators in each group [14]. Several studies on the elderly population have used LCA to integrate health indicators from physical and psychological dimensions and to identify heterogeneous health groups in other parts of the world [7,15,16,17]. However, to the best of our knowledge, studies on the heterogeneous and multidimensional health groups of the elderly Chinese population by person-centered approaches remains limited.

Previous researches on the elderly population have offered some insights into the differences in healthcare utilization. The elderly population with a high level of comorbidities required more hospitalizations and visits to the emergency department rather than to clinics [18]. Moreover, the frail elderly people need more medical specialty follow-ups, and hence have a higher likelihood of requiring a caregiver [19]. By contrast, the elderly people having social support from family were less likely to use outpatient services [20]. The studies that identified heterogeneous health groups for elderly people also emphasized that the patterns of healthcare services utilization varied across different health groups [7,15,16,17]. However, these studies considered only the physical and psychological health dimensions, with neglect of social health dimension, and the impact of multidimensional health groups on healthcare utilization was not well documented.

The objective of our study is to integrate multidimensional health indicators to identify the heterogeneity of health status among elderly Chinese people by latent health groups. Further, the effects of multidimensional health groups and socio-demographic factors on healthcare utilization were assessed by Andersen’s model.

## 2. Materials and Methods 

### 2.1. Data Source and Study Population

We used data from the Chinese Longitudinal Healthy Longevity Survey (CLHLS) in 2014. The CLHLS is a nationally representative longitudinal survey of the elderly population of China. The detailed information about the survey design has been reported in the previous study [21]. After excluding the elderly participants aged <65 years or those with missing health indicators, this study included 2981 participants for the analyses of multidimensional health groups. From which, 1974 participants with complete information on healthcare utilization and socio-demographic characteristics were included in the analyses of impact factors on healthcare utilization.

### 2.2. Measures

Andersen’s behavioral model is one of the most frequently applied frameworks for the study of healthcare utilization that outlines the three determinants: predisposing, enabling, and need factors [22]. Based on this, we evaluated the effects of health groups, as need factors, and associated socio-demographic factors, as predisposing and enabling factors, on healthcare utilization.

#### 2.2.1. Need Factors

To obtain the multidimensional health groups as need factors, this study organically integrated seven health indicators from physical, psychological, and social dimensions.

The physical dimension of health status was evaluated by chronic conditions and functional disabilities. Twelve common, chronic, and non-communicable diseases were selected, including hypertension, heart disease, stroke, dementia, Parkinson’s disease, epilepsy, diabetes, respiratory disorders, cancer, arthritis, cataract, and glaucoma. These were further classified into seven chronic conditions by International Statistical Classification of Diseases and Related Health Problems 10th Revision, which included cardiovascular, neurological, endocrine, respiratory, cancer, musculoskeletal, and sensorial conditions. The measures of chronic conditions were defined as “no chronic condition”, “one chronic condition”, or “two and over chronic conditions”. Functional disabilities were measured by two health indicators: ADLs and instrumental activities of daily living (IADLs). Activities of ADLs or IADLs requiring any assistance were defined as “with difficulties”. The measure of ADLs was assessed as “no difficulty” or “with difficulties”. The measure of IADLs was defined as “0–2 activities”, “3–4 activities”, or “≥5 activities” with difficulties.

The psychological dimension of health status was evaluated by the cognitive problem and depressive symptom. The cognitive problem was measured using the Chinese version of the mini-mental state examination (MMSE; 0–30 scores) and educational attainment. The education-adjusted MMSE cut-points were: scores ≤19 for those without education, scores ≤22 for those with ≤6 years of education, and scores ≤26 for those with ≥7 years of education [8]. Participants scoring below the education-adjusted MMSE cut-points were regarded as having the cognitive problem. The depressive symptom was evaluated from two questions: “In the last 12 months, have you felt sad, blue, or depressed for two weeks or more?” and “In the last 12 months, have you lost interest in things like hobbies, work, or activities that usually give you pleasure?”. Participants with any “yes” response to these questions were regarded as having a depressive symptom [23].

The social dimension of health status was composed of structural and functional relationships [24]. The structural relationships included four items with 0–4 scores: marital status (1 = in marriage, 0 = not in marriage), close children (1 = have close children, 0 = otherwise), playing cards and/or mah-jongg (1 = at least once a month or more, 0 = otherwise), and attending social activities (1 = at least once a month or more, 0 = otherwise). Those with 0–2 scores were defined as “lacking structural relationship”. The functional relationships were evaluated using three questions: “To whom do you usually talk most frequently in daily life?”, “To whom do you talk first when you need to tell something of your thoughts?”, and “Who do you ask first for help when you have problems/difficulties?”. For each question, participants could select three from ten options of relationships (without repetition). The first, second, and third selections were defined as 3, 2, and 1 score, respectively. Only the selections among kinship and friends/neighbors were counted. The scores of the three questions ranged from 0–18. Scores below 12 were considered as lacking a functional relationship.

#### 2.2.2. Predisposing and Enabling Factors

Socio-demographic characteristics associated with predisposing and enabling factors in Andersen’s behavioral model were evaluated in this study. The predisposing factors included age (65–79 years, ≥80 years), gender (male, female), marital status (not in marriage, in marriage), living arrangement (alone, with others), education (illiterate, literate, or primary school, junior high and above), and main occupation before age 60 years (agriculture, professional/management occupation, and others). The enabling factors included residence area (rural, urban), annual household income per capita (divided into quartiles: <7000 yuan, 7000–20,000 yuan, 20,000–40,000 yuan, and >40,000 yuan), and three main health insurance: Urban Employee Basic Medical Insurance (UE-BMI), Urban Resident Basic Medical Insurance (UR-BMI), and New Rural Cooperative Medical Scheme (NRCMS).

#### 2.2.3. Healthcare Utilization

The outcome variables in this study included outpatient and inpatient expenditure during the last year, which was assessed based on two questions: “How much did you spend on outpatient cost in last year?” and “How much did you spend on inpatient cost in last year?”

### 2.3. Statistical Analyses

The statistical analyses in this study involved three steps. The first step was to identify the health groups by LCA. In the LCA model, individuals were classified into unobservable health groups. With regard to the characteristics of health indicators, individuals within a group were similar to one another but different from those in other groups. The LCA estimated two types of parameters: conditional health indicators probabilities and health latent group probabilities [25]. Conditional health indicators probabilities specified the associations between observed health indicator and unobservable health groups. Health latent group probabilities represented individuals’ relative probabilities of being assigned to each group. The optimal model was obtained by increasing the number of identified health groups until no improvement was observed. In our study, LCA models with two to five groups were conducted and compared by the following criteria: Bayesian information criterion (BIC), adjusted BIC (aBIC), and consistent Akaike’s information criterion (cAIC) [26]. The smaller values of the above criteria indicated better model performance. 

The next step was to examine the differences in healthcare utilization among health groups by nonparametric tests. Chi-squared test was used for categorical variables, and the Shapiro–Wilk test, Levene’s test, Kruskal–Wallis test, and Steel Dwass test were used for skewed continuous variables. The last step was to construct a two-part model to analyze the impact of need, predisposing, and enabling factors on healthcare utilization. The logistic model was adopted in the first part of the two-part model, and the ordinary least square model with the log-transformed was used in the second part.

All statistical analyses were performed using R software (version 3.5.3; R Foundation for Statistical Computing, Vienna, Austria). A *p*-value below 0.05 was regarded to be statistically significant.

## 3. Results

### 3.1. Multidimensional Health Groups of Elderly Adults

For 1974 elderly participants with complete information, the characteristics of need, predisposing, and enabling factors of health utilization are presented in Table 1. In terms of the need factor, only two out of five participants had no common chronic diseases. About 10.7% of participants had difficulties in ADLs, while 16.5% had ≥5 activities with difficulties in IADLs. Over 14% of elderly participants had psychological problems. More than half the population lacked social relationships. With regards to predisposing factors, more than half the participants were over 80 years old, and 52.6% were male in the study population. Nearly half the elderly people were in marriage, and 53.5% were literate or had received the formal education. Nearly seven out of ten participants worked in the agricultural sector before the age of 60 years. For enabling factors, 47.8% of the study population lived in rural areas, and nearly one out of five lived alone. Nearly a quarter of the elderly had household income below 7000 yuan (USD 1077; CNY 6.5 per USD). Those enrolled in UE-BMI, UR-BMI, and NRCMS insurance scheme were 13.5%, 8.1%, and 69.4%, respectively. 

Table 2 shows the performance of LCA models with two to five groups for 2981 elderly participants without missing any health indicators. The model with four groups obtained the least BIC (24065.84), aBIC (23941.92), and cAIC (24104.84), and hence was adopted as the final model in this study.

The health groups were labeled according to the characteristics of conditional health indicators probabilities (λ; Table 3). The first group (*N* = 311, 10.4%) had the highest probabilities of lacking a structural relationship (λ = 0.810) and lacking a functional relationship (λ = 0.766). Hence, this group was labeled as “Lacking Socialization” (LS). The second group (*N* = 498, 16.7%) had the highest probabilities of chronic conditions (λ = 0.415 for one chronic condition and λ = 0.399 for two or more chronic conditions) and a depressive symptom (λ = 0.290). Thus, we labeled this group as “High Comorbidity” (HC). With the highest probabilities of ADLs difficulties (λ = 0.750), ≥5 IADLs difficulties (λ = 1.000) and a cognitive problem (λ = 0.260), the third group (*N* = 231, 7.8%) was labeled as “Severe Disability” (SD). The last group, labeled as “Relative Health” (RH), was the largest (*N* = 1941, 65.1%) and identified with the lowest probability of ADLs difficulties (λ = 0), ≥3 IADLs difficulties (λ = 0), a cognitive problem (λ = 0.011), and lacking a structural relationship (λ = 0.277).

### 3.2. Healthcare Utilization Among Different Health Groups

The results of non-parametric tests indicated that different patterns of utilization of healthcare services existed among the four health groups (Table 4). Overall, 1468 (74.4%) elderly participants used the outpatient services, which cost an average of CNY 2272.11 (USD 349.56). Meanwhile, 566 (28.7%) participants went to the hospital and their average expenditures were CNY 9165.38 (USD 1410.06) in the past year. A significant difference was found in the use of outpatient and inpatient services among the health groups (*p* < 0.001). Elderly participants from the HC group were more likely to take outpatient visits costing the highest expenditure, while those from the SD group were more likely to be hospitalized. The differences in inpatient expenditure among different health groups were not significant.

### 3.3. The Effects of Health Groups and Other Influencing Factors on Healthcare Utilization

The results of two-part models on healthcare utilization are as shown in the Table 5. In the first part model on the likelihood of healthcare utilization, elderly participants were more likely to use both outpatient and inpatient services in the HC (OR = 1.968 and 2.264, respectively) and SD (OR = 1.895 and 2.707, respectively) groups than the RH group. Additionally, the age (*p* < 0.001) and education (*p* = 0.02) were significantly associated with the likelihood of use of outpatient services in elderly participants. When controlling for health group as the need factor, elderly participants aged 80 years and over were less likely to use both outpatient and inpatient service (OR = 0.724 and 0.751, respectively). Likewise, those with an education level of junior high and above showed less tendency to use the outpatient services (OR = 0.641). 

For the second part model on the amount of expenditure, elderly participants in the HC and SD groups accounted for higher outpatient expenditure (Coef. = 0.586 and 0.612, respectively), and those in the LS groups resulted in higher inpatient expenditure (Coef. = 0.529). For predisposing and enabling factors, age, gender, marital status, main occupation before the age 60 years, and residence area had significant effects on outpatient expenditure, while the health insurance scheme significantly impacted both, the outpatient and inpatient expenditure. Older and male participants accounted for lower outpatient expenditure (Coef. = −0.245 and −0.166, respectively). Meanwhile, those in marriage, with professional/managerial or other non-agriculture occupations, younger than 60 years, and living in urban areas cost higher inpatient expenditure (Coef. = 0.204, 0.540, 0.325, and 0.214, respectively). Elderly adults enrolled in NRCMS showed a tendency to incur less outpatient and inpatient costs (Coef. = −0.504 and −0.573, respectively).

## 4. Discussion

We examined the heterogeneity of health status among the elderly Chinese based on multidimensional health, and four health groups were delineated using LCA. The findings of our study strengthened the understanding of the patterns of healthcare utilization among elderly Chinese. Moreover, it provided a more comprehensive measurement on the relationships between multidimensional health groups of the elderly population and their utilization of healthcare than earlier studies [7,15,27].

In this study, the social dimension of health status was integrated and identified as a distinctive feature of LS health group. Although this group had a low likelihood of utilizing the inpatient services, the participants in this group who were hospitalized, eventually incurred a high expenditure. The significant effect of the LS group on inpatient expenditure was also verified by Andersen’s model. Elderly participants in the LS group lacked social support and sought fewer healthcare services on getting sick [28]. The delayed treatment may lead to the worsening of physical and psychological health status, as some studies revealed [24,29]. This may eventually result in additional expenditures when hospitalization is needed. Active engagement in socialization has been an indispensable part of the active ageing as promoted by the WHO [30]. Thus, more attention must be given to the health status of the elderly about the possible changes in the social dimension and its effects on the healthcare expenditures.

Our study found that elderly people in the HC group strained healthcare services heavily. These elderly participants suffering from multiple chronic diseases were more likely to utilize the outpatient services and incur the highest outpatient expenditure, agreeing with the previous studies [31,32,33]. For convenience, these elderly participants tended to take outpatient visits to acquire pharmaceutical treatments and primary care consultations [34,35]. Owing to the lack of well-qualified medical workers in China, the primary health center (PHC) at the community level is compromised in dealing with the management of chronic diseases [36]. Previous research has found that introducing pharmacist management of patients in the outpatient services can help in reducing the burden of PHC [37]. This may contribute to chronic diseases management for the elderly population.

The remarkable characteristics for the elderly participants in the SD group were the high probability of disabilities and cognitive problem, which were consistent with some previous studies indicating that cognitive problem may be associated with disabilities [38,39]. Our study showed that these elderly participants had a higher likelihood of healthcare utilization and high outpatient expenditure. These results were similar to earlier studies on elderly people seeking healthcare services [32,40]. It has been pointed out that the traditional family care may not be sufficient to manage the elderly people with disabilities, due to the need of immediate medical services and the changing attitude on filial responsibilities [4,41]. It further needs to be supplemented with integrated and sustainable long-term care based on institution and community [42].

In terms of predisposing factors, results of Andersen’s model revealed that age had no and significantly negative effect on inpatient and outpatient expenditures, respectively. Inconsistent with our findings, some previous literature indicated that the effect of age on healthcare expenditure was not significant for elderly people, after controlling for proximity to death [43,44,45]. With the increasing life expectancy and the process of healthy ageing, the effect of age on healthcare utilization is worthy of further discussion. For the enabling factors, the findings of this study indicated that NRCMS had significantly negative effects on healthcare expenditure. Contrastingly, previous elderly Chinese studies that were without regard to the heterogeneity of health did not arrive at this finding [46,47]. For the patients wanting to get high reimbursement from NRCMS, the insufficient use of inpatient services may result in the illusion of a decrease in expenditure [40,48]. To eliminate the disparities in health insurance, the Chinese government announced a blueprint of consolidating NRCMS and UR-BMI into a unified basic health insurance system in early 2016 [49,50]. Its effect on healthcare utilization for rural elderly people is worth further study.

After controlling for health groups, we found the effects of gender, marital status, and primary occupation before 60 years, as predisposing factors, and residential area, as enabling factors were not significant on inpatient utilization by Andersen’s model. Further, the inpatient expenditures were principally determined by health groups among the elderly population. However, these factors still had a significant impact on outpatient expenditure. These findings indicated that the equality and efficiency issues about outpatient utilization deserved more attention than inpatient utilization in China. To relieve the outpatient workload in hospitals, the Chinese government was processing to establish the community health centers which could provide preventive and primary care [51]. However, due to the shortage of resources and poor quality, patients with common diseases remained distrustful towards PHC and continued to access the hospital outpatient services [52,53]. Introducing multidimensional health groups could deepen our understanding of the impact of socio-demographic factors on healthcare utilization for elderly people. These findings shed light on the implications of health policies aiming to provide equitable and efficient healthcare services. 

Our study has several limitations. Firstly, this study was based on participants’ self-reported information, which may suffer from measurement error, such as the participants’ status of diseases, healthcare expenditure, etc. Secondly, this study adopted the cross-sectional data, and it could be worth further discussion on the causal effects of multidimensional health groups on healthcare utilization by longitudinal data. Third, this study adopted a limited set of variables on healthcare utilization. Further researches on more healthcare utilization information could be fruitful.

## 5. Conclusions

Based on the multidimensional health of the elderly population in China, four health groups were identified using person-centered approach of LCA. The results of this study indicated significant effects of health groups on both, the likelihood of healthcare utilization and subsequent expenditures by Andersen’s model. After controlling for health groups, different effects of socio-demographic factors on healthcare utilization were found. This study strengthened our understanding of complex health status for elderly people and provided a more comprehensive assessment of the associations between multidimensional health groups and healthcare utilization. Further, it also provided evidence of the improvement of the allocation of the healthcare resource targeted for different health groups in the ageing population.

## Figures and Tables

**Table 1:** Characteristics of need, predisposing, and enabling factors of health utilization for elderly adults.

Variables	*N*	%
Need factors		
Physiologic health status		
Number of chronic conditions		
0	824	41.7
1	722	36.6
≥2	428	21.7
ADLs difficulties	212	10.7
IADLs difficulties		
0–2	1399	70.8
3–4	242	12.3
≥5	333	16.9
Psychological health status		
Cognitive problem	93	4.7
Depressive symptom	283	14.3
Social health status		
Lacking structural relationship	864	43.8
Lacking functional relationship	995	50.4
Predisposing factors		
Age		
65–79	920	46.6
≥80	1054	53.4
Gender		
Female	936	47.4
Male	1038	52.6
Marital status		
Not in marriage	975	49.4
In marriage	999	50.6
Education		
Illiterate	918	46.5
Literate or primary school	747	37.8
Junior high and above	309	15.7
Occupation before 60		
Agriculture	1394	70.6
Professional/managerial	164	8.3
Others	416	21.1
Enabling factors		
Residence area		
Rural area	943	47.8
Urban area	1031	52.2
Living status		
Alone	380	19.3
With others	1594	80.7
Household income		
Lower than 7000 yuan	488	24.7
7000–20,000 yuan	385	19.5
20,000–40,000 yuan	501	25.4
Higher than 40,000 yuan	600	30.4
Health insurance		
UE-BMI	266	13.5
UR-BMI	160	8.1
NRCMS	1370	69.4

*N* = 1974 with complete information. Abbreviations: ADLs, activities of daily living; IADLs, instrumental activities of daily living; UE-BMI, Urban Employee Basic Medical Insurance; UR-BMI, Urban Resident Basic Medical Insurance; NRCMS, New Rural Cooperative Medical Scheme.

**Table 2:** Performance of latent class analysis (LCA) models with 2–5 groups.

Indexes	2 Groups	3 Groups	4 Groups	5 Groups
BIC	24,102.99	24,088.81	24,065.84	24,104.05
aBIC	24,042.62	23,996.66	23,941.92	23,948.36
cAIC	24,121.99	24,117.81	24,104.84	24,153.05

Abbreviations: LCA, latent class analysis; BIC, Bayesian information criterion; aBIC, adjusted Bayesian information criterion; cAIC, consistent Akaike information criterion.

**Table 3:** Conditional probabilities of health indicators in each health groups for elderly adults.

Variables	Sample Proportion (*N* = 2981)	Lacking Socialization (*N* = 311)	High Comorbidity (*N* = 498)	Severe Disability (*N* = 231)	Relative Health (*N* = 1941)
Physiologic health status					
Number of chronic conditions					
0	0.416	0.633	0.186	0.355	0.472
1	0.377	0.334	0.415	0.363	0.374
≥2	0.207	0.033	0.399	0.282	0.154
ADLs difficulties	0.107	0.036	0.166	0.750	0.000
IADLs difficulties					
0–2	0.708	0.360	0.519	0.000	1.000
3–4	0.127	0.324	0.328	0.000	0.000
≥5	0.165	0.316	0.153	1.000	0.000
Psychological health status					
Cognitive problem	0.051	0.091	0.045	0.260	0.011
Depressive symptom	0.136	0.037	0.290	0.216	0.078
Social health status					
Lacking structural relationship	0.433	0.810	0.430	0.797	0.277
Lacking functional relationship	0.535	0.766	0.380	0.604	0.534

Abbreviations: ADLs, activities of daily living; IADLs, instrumental activities of daily living.

**Table 4:** Healthcare utilization among different health groups.

Health Groups and Nonparametric Tests	Users				Expenditure (CNY)			
	Outpatient		Inpatient		Outpatient		Inpatient	
	*N*	%	*N*	%	Mean	Std Dev	Mean	Std Dev
Overall	1468	74.4	566	28.7	2272.11	5208.59	9165.38	13,787.25
Lacking Socialization	142	66.4	37	17.3	1143.66	1692.30	9521.08	11,635.56
High Comorbidity	283	84.0	143	42.4	3653.75	7770.13	10513.50	14,576.89
Severe Disability	124	82.1	66	43.7	3467.64	7616.05	8646.97	9834.12
Relative Health	919	72.3	320	25.2	1859.70	3930.25	8628.73	14,350.34
χ2 test	<0.001		<0.001					
Shapiro–Wilk test					<0.001		<0.001	
Levene’s Test					<0.001		0.76	
Kruskal–Wallis Test					<0.001		0.07	
Steel Dwass test					LS < RH < HC, SD			

*N* = 1974 with complete information. Abbreviations: CNY, Chinese yuan; Std Dev, standard deviation; LS, Lacking Socialization; HC, High Comorbidity; SD, Severe Disability; RH, Relative Health.

**Table 5:** Results of the two-part model on healthcare utilization for elderly adults.

Variables	Part 1				Part 2			
	Outpatient		Inpatient		Outpatient		Inpatient	
	OR	*p*-Value	OR	*p*-Value	OR	*p*-Value	OR	*p*-Value
Need factors								
Health group								
Relative Health	ref		ref		ref		ref	
Lacking Socialization	0.869	0.43	0.769	0.21	0.061	0.64	0.529	0.020
High Comorbidity	1.968	<0.001	2.264	<0.001	0.586	<0.001	0.104	0.41
Severe Disability	1.895	0.006	2.707	<0.001	0.612	<0.001	0.155	0.38
Predisposing factors								
Age								
65–79	ref		ref		ref		ref	
≥80	0.724	0.008	0.751	0.015	−0.245	0.002	0.015	0.90
Gender								
Female	ref		ref		ref		ref	
Male	0.817	0.10	0.942	0.61	−0.166	0.036	−0.142	0.24
Marital status								
Not in marriage	ref		ref		ref		ref	
In marriage	1.041	0.77	1.120	0.39	0.204	0.024	0.233	0.08
Education								
Illiterate	ref		ref		ref		ref	
Literate or primary school	0.880	0.32	0.980	0.87	0.107	0.21	0.188	0.14
Junior high and above	0.641	0.019	0.864	0.43	0.015	0.91	0.079	0.68
Main occupation before age 60								
Agriculture	ref		ref		ref		ref	
Professional/managerial	1.140	0.61	1.369	0.19	0.540	0.001	0.258	0.27
Others	1.232	0.20	1.254	0.13	0.325	0.001	0.130	0.39
Enabling factors								
Residence area								
Rural area	ref		ref		ref		ref	
Urban area	1.141	0.25	1.135	0.26	0.215	0.005	0.062	0.59
Living status								
Alone	ref		ref		ref		ref	
With others	1.086	0.60	0.940	0.70	−0.082	0.44	0.034	0.83
Household income								
Lower than 7000 yuan	ref		ref		ref		ref	
7000–20,000 yuan	0.948	0.73	0.949	0.74	0.131	0.23	−0.156	0.33
20,000–40,000 yuan	1.051	0.75	0.982	0.91	0.172	0.10	0.164	0.30
Higher than 40,000 yuan	1.192	0.28	1.014	0.93	0.188	0.08	0.131	0.41
Health Insurance								
UE-BMI	1.203	0.45	0.957	0.83	−0.195	0.17	0.099	0.62
UR-BMI	1.145	0.62	0.951	0.83	−0.131	0.41	0.065	0.78
NRCMS	0.883	0.54	0.951	0.79	−0.504	<0.001	−0.573	0.003

*N* = 1974 with complete information. Abbreviations: OR: odds ratio; Coef.: Coefficient; UE-BMI, Urban Employee Basic Medical Insurance; UR-BMI, Urban Resident Basic Medical Insurance; NRCMS, New Rural Cooperative Medical Scheme.
